# Quantifying Renin-Angiotensin-System Alterations in COVID-19

**DOI:** 10.3390/cells10102755

**Published:** 2021-10-14

**Authors:** Fabrizio Pucci, Filippo Annoni, Robson Augusto Souza dos Santos, Fabio Silvio Taccone, Marianne Rooman

**Affiliations:** 1 3BIO—Computational Biology and Bioinformatics, Université Libre de Bruxelles, 1050 Brussels, Belgium; Fabrizio.Pucci@ulb.be; 2 (IB)^2^—Interuniversity Institute of Bioinformatics in Brussels, 1050 Brussels, Belgium; 3 Department of Intensive Care, Hôpital Erasme, Université Libre de Bruxelles, 1070 Brussels, Belgium; Filippo.Annoni@erasme.ulb.ac.be (F.A.); fabio.taccone@ulb.ac.be (F.S.T.); 4 Department of Physiology and Biophysics, Federal University of Minas Gerais, Belo Horizonte 31270, MG, Brazil; robsonsant@gmail.com

**Keywords:** SARS-CoV-2, spike protein, ACE2, Angiotensin II, Angiotensin-(1-7), RAS dysregulation, RAS-targeting drugs

## Abstract

The renin-angiotensin system (RAS) plays a pivotal role in a wide series of physiological processes, among which inflammation and blood pressure regulation. One of its key components, the angiotensin-converting enzyme 2, has been identified as the entry point of the SARS-CoV-2 virus into the host cells, and therefore a lot of research has been devoted to study RAS dysregulation in COVID-19. Here we discuss the alterations of the regulatory RAS axes due to SARS-CoV-2 infection on the basis of a series of recent clinical investigations and experimental analyzes quantifying, e.g., the levels and activity of RAS components. We performed a comprehensive meta-analysis of these data in view of disentangling the links between the impaired RAS functioning and the pathophysiological characteristics of COVID-19. We also review the effects of several RAS-targeting drugs and how they could potentially help restore the normal RAS functionality and minimize the COVID-19 severity. Finally, we discuss the conflicting evidence found in the literature and the open questions on RAS dysregulation in SARS-CoV-2 infection whose resolution would improve our understanding of COVID-19.

## 1. Introduction: Normal RAS Regulation

The systemic renin-angiotensin system (RAS) has been extensively studied in the last decades, due to its key regulatory roles in cardiovascular physiology [1,2]. Its dysfunction has been related to a wide range of cardiovascular diseases including hypertension and atherosclerosis, and cardiac dysfunctions, including myocardial hypertrophy and arrhythmia [3,4]. More recently, the involvement of RAS in a wide spectrum of other diseases has become evident. For example, RAS is altered in pulmonary diseases such as asthma, acute respiratory distress syndrome (ARDS) and chronic obstructive pulmonary disease, as well as in inflammatory responses associated with liver and renal disorders [5,6,7].

**Figure 1:**
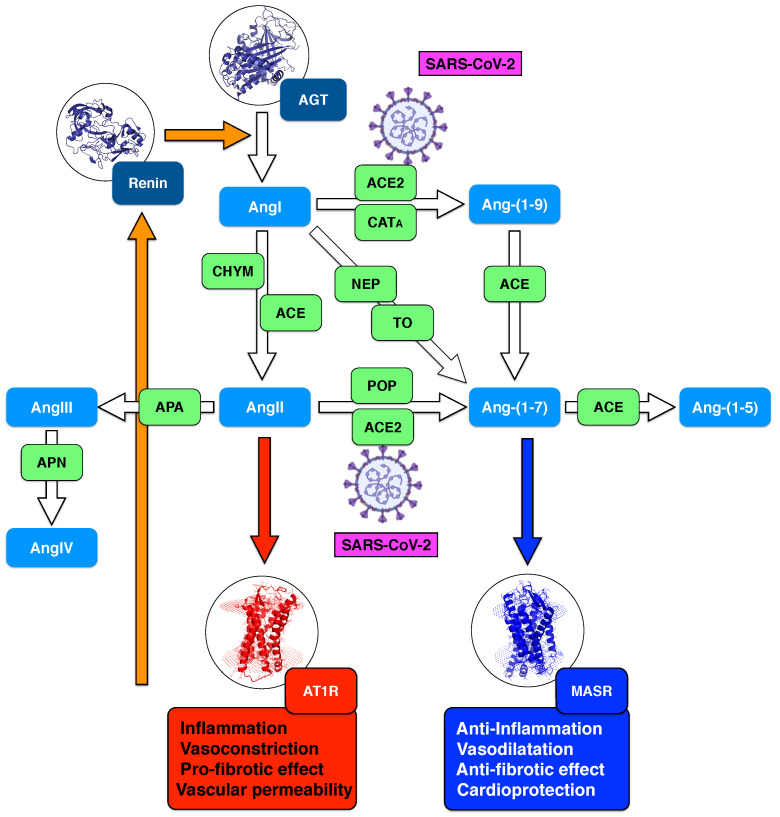
Schematic representation of systemic RAS. The peptides belonging to RAS (AngI, AngII, Ang-(1-5), Ang-(1-7), Ang-(1-9), AngIII, AngIV) are in light blue boxes; renin produced by juxtaglomerular kidney cells and angiotensinogen produced by the liver are in dark blue boxes; the enzymatic actors of RAS are in green boxes: angiotensin converting enzyme (ACE), angiotensin converting enzyme 2 (ACE2), neprilysin (NEP), chymase (CHYM), cathepsin A (CAT${}_{A}$), thimet oligopeptidase (TO), aminopeptidase A (APA), aminopeptidase N (APN) and prolyloligopeptidase (POP). AngII type 1 receptor (AT1R) is in a red box, with the classical AngII/AT1R axis indicated with a red arrow; the MAS receptor (MASR) is in a deep blue box with the counter-regulatory axis Ang-(1-7)/MASR indicated with a deep blue arrow. The RAS feedback loop is shown with orange arrows. SARS-CoV-2, which acts on ACE2 through the binding of its spike protein, is indicated in magenta.

A schematic representation of the enzymatic reactions involved in systemic RAS is shown in Figure 1. In brief, in response to decreased blood pressure, or to decreased sodium load in the distal convoluted tubule, juxtaglomerular kidney cells secret renin. This protease cleaves the ten N-terminal amino acids of angiotensinogen, an α-2-globulin synthesized in the liver, to form the decapeptide angiotensin-I (AngI). AngI is physiologically inactive, and is cleaved into a series of smaller peptides:

- angiotensin 1-9 (Ang-(1-9)) nonapeptide via angiotensin-converting enzyme 2 (ACE2) or cathepsin A (CAT${}_{A}$);
- angiotensin-II (AngII) octapeptide via angiotensin-converting enzyme (ACE) or chymase (CHY);
- angiotensin-(1-7) (Ang-(1-7)) heptapeptide via neprilysin (NEP) or thimet oligopeptidase (TO).

Ang-(1-7) is also obtained by cleavage of AngII by ACE2 and prolyl oligopeptidase (POP), and of Ang-(1-9) by ACE. It is further cleaved to angiotensin-(1-5) (Ang-(1-5)) via ACE. Angiotensin-III (AngIII) is the result of the cleavage of AngII by aminopeptidase A (APA), and is in turn cleaved to angiotensin-IV (AngIV) by aminopeptidase N (APN). Note that other peptides such as angiotensin-(1-4), (3-7), (5-7), (3-4) are found in the enzymatic cascades of Ang-(1-7) and AngII, but their functions are not yet fully understood.

The two main effectors in RAS are AngII and Ang-(1-7). Their biological effects, mediated by their binding to AngII type 1 receptor (AT1R) and MAS receptor (MASR), respectively, are completely different. The ACE/AngII/AT1R axis, also known as the classical RAS axis, promotes vasoconstriction, cell growth and fibrosis, and increases inflammation and oxidative stress [8]. In contrast, the ACE2/Ang1–7/MASR axis, called non-classical or counter-regulatory RAS axis, acts in opposition with the classical axis by promoting vasodilation, decreasing inflammation and oxidative stress and inhibiting cell growth [9,10]. More and more evidence suggests that the counter-balancing effects of these axes play a central role in cardiovascular physiology. Moreover, the dysregulation of this balance appears to be directly related to disease conditions [11]. It is thus important to better understand it in order to shed light on the pathophysiology of cardiovascular disorders and to design new therapeutic strategies [12,13,14].

While this simplified two-axes picture of RAS is quite appealing, the full cascade of RAS enzymatic reactions is much more complex. First, AngII can also bind to AngII type 2 receptor (AT2R), which is mainly expressed in foetal and embryonic tissues. In adults, AT2R is observed under pathophysiological conditions, for example in neointima after vascular injury, and in myocardial tissues after ischemia [15]. This defines the additional ACE/AngII/AT2R axis that leads to effects that are different, and sometimes antagonistic, to those caused by the binding of AngII to AT1R [16]. Secondly, AngIII also appears to be a RAS effector, which binds to both AT1R and AT2R, thereby creating yet additional axes, APA/AngIII/AT1R and APA/AngIII/AT2R [17]. Another important axis involves the recently discovered vasoactive peptide called alamandine, that is generated via decarboxylation of Ang-(1-7) or via ACE2 catalytic action on angiotensin A (AngA), a peptide which differs from AngII by one amino acid. Alamandine binds to the MAS-related receptor D (MrgD), thus defining another counter-regulatory axis with a protective action similar to ACE2/Ang-(1-7)/MASR [18]. In addition, other, smaller, angiotensin peptides are likely to play physiological roles even though they are not fully known at the moment [19].

Another level of complexity has recently been highlighted: in addition to the systemic RAS which we mainly focus on in this review, recent findings point towards the importance of local, organ- and tissue-based, RAS, which seems to partially act independently from systemic RAS and to have distinct physiological roles [20,21]. These different local RASs are of course much more difficult to analyze experimentally and clinically than systemic RAS. Indeed, their components are difficult to reach and characterize, in contrast to systemic RAS components that are present in the plasma. ACE2 enzymes, in particular, exist in two forms, a circulating soluble form which is part of the systemic RAS, and a membrane-bound form which belongs to the local RAS and is mainly found on the surface of type II alveolar epithelial cells, epithelial cells of oral mucosa and myocardial cells [22]. Cellular mechanisms called shedding regulate the conversion between the soluble and membrane-bound forms [23], thus demonstrating that local and systemic RAS are not totally independent.

During the past two years, much attention has been devoted to studying the role of RAS in SARS-CoV-2 infection [24,25,26]. Indeed, the spike protein of this virus interferes with RAS, as it binds to the membrane-bound form of ACE2 in order to enter host cells [27,28]. It has therefore been speculated that RAS dysregulation in COVID-19 is connected to the severity of the disease and to its clinical manifestations such as hyper-inflammation, ARDS and thrombotic complications [29,30].

## 2. RAS Dysregulation in the First SARS-CoV Infections

The idea that RAS dysregulation plays a role in coronaviral infections and is related to one of their clinical manifestations, i.e., ARDS, has been suggested almost twenty years ago, in 2003, when the first SARS-CoV infections were identified [31,32].

The key role of ACE2 in RAS dysregulation has been demonstrated a few years later through in vitro analyses and in vivo animal experiments [31,33,34]. ACE2 was shown to have a protective role in lung injury and to act in opposition to ACE by downregulating AngII levels and thereby mitigating its pro-inflammatory effects. Indeed, ACE2 knockout mice were seen to have worsened oxygenation, higher levels of inflammation markers and increased lung oedema in ARDS induced by acid aspiration or sepsis [33]. Instead, the administration of recombinant ACE2 was able to rescue these adverse pathogenic signatures.

In parallel, data from SARS-CoV infected mice indicated that the spike protein of the virus binds to ACE2 and reduces ACE2 expression, and that injection of spike protein into mice worsens the severity of lung failure [34]. This confirmed the crucial implication of ACE2, not only in acid aspiration- or sepsis-induced ARDS, but also in ARDS caused by SARS-CoV infection.

These observations on animal models naturally led to think about the administration of recombinant human ACE2 (rhACE2) as a possible treatment against SARS-CoV infections in humans and, more generally, against ARDS. Indeed, rhACE2 was argued to have a dual role in fighting coronaviral infections: binding to the viral spike protein and thus blocking the spread, entrance into the host cells and replication of SARS-CoV, and simultaneously downregulating AngII levels and therefore protecting SARS-CoV infected patients from lung failure and attenuating strong inflammatory response. However, these hypotheses proved to be far too optimistic, at least for humans, as revealed by data from the new SARS-CoV-2 infections discussed in the next sections.

## 3. RAS Dysregulation in Current SARS-CoV-2 Infections: New Data and Hypotheses

Since the end of 2019, when SARS-CoV-2 appeared for the first time in Wuhan [29,35,36], a lot of research has been devoted to understanding and characterizing the molecular basis of the infection. ACE2 was soon suggested to be the mediator of the viral invasion of the host cells through its binding with the spike protein of SARS-CoV-2, with the concomitant downregulation of ACE2-involving pathways [37,38]. These findings were largely inspired by the similarity with previous SARS-CoV infections [31,33,34]. It has then been speculated that ACE2 downregulation impacts on the expression of all other components of RAS and leads to RAS dysregulation connected to the severity of COVID-19 [39,40,41].

**Figure 2:**
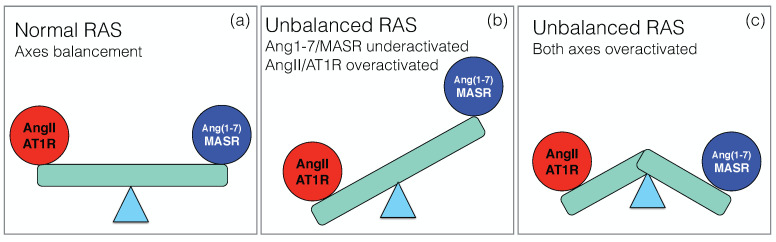
Schematic representation of the two main RAS axes ACE/AngII/AT1R and ACE2/Ang-(1-7)/MASR in: (**a**) controls and (**b**,**c**) CoViD-19 patients with two possible dysregulated RAS scenarios.

Under healthy conditions, the two main axes of RAS, the pro-inflammatory ACE/ AngII/AT1R axis and the counter-regulatory ACE2/Ang1–7/MASR axis, balance their effects in order to maintain normal physiological functions, as schematically depicted in Figure 2a. Upon SARS-CoV-2 infection, this balance breaks down, but the way in which RAS dysregulation occurs is still unclear, and different scenarios have been proposed.

According to a first scenario, the virus is internalized through its binding with the membrane-bound form of ACE2, which causes a decrease of the amount of ACE2 on the host cell surface and affects the equilibrium between the two RAS axes. On the one hand, the AngII level increases, with consequent activation of the classical AngII/AT1R signaling cascade [14,42]. This directly leads to a strong pro-inflammatory and fibrotic response [43] likely to drive acute lung injury. On the non-classical RAS axis, the downregulation of ACE2 results in a loss of Ang-(1-7) and thus in the inhibition of its downstream signaling pathways with their protective role [10,44]. All the beneficial outcomes of this axis, including its anti-inflammatory, anti-oxidative and anti-fibrotic effects, are thus severely compromised and further worsen pulmonary distress and vascular dysfunction.

The picture that emerges here is thus an overactivation of the classical axis and underactivation of the non-classical axis, as shown schematically in Figure 2b. It is supported by a series of papers [24,45,46,47,48,49,50]. Since local RAS plays a key role in a wide series of physiological processes in different organs, this imbalance has been speculated to explain the multi-organ failure associated to severe COVID-19 and put in relation with the different pathophysiological characteristics of the disease. They include not only ARDS [51], but also heart failure [52], renal dysfunction [53], defective coagulation with an increase of thrombotic events [54,55] and neurological manifestations [56].

A second scenario has been proposed [57,58], in which the non-classical RAS axis is overactivated rather than underactivated (Figure 2c). It is based on the idea that the internalization of the virus into the host cells is not the only key event, but that another crucial event is ACE2 shedding: upon binding of the spike protein to the membrane-bound form of ACE2 (mACE2), the ACE2 ectodomain is detached from the cell membrane and released into the serum. This circulating, soluble ACE2 (sACE2) is also catalytically active and able to bind to the spike protein.

Note that ACE2 shedding is a cellular mechanism which also occurs under healthy conditions and is mainly driven by a metalloproteinase, the tumor necrosis factor (TNF)-α converting enzyme (TACE, also known as ADAM17) [23]. ADAM17 is expressed in a series of tissues, such as lungs, kidney, small intestine and heart, and cleaves a variety of membrane-anchored cytokines [59]. Its overexpression has been related to several pathophysiological conditions that range from vascular dysfunctions to pulmonary disease and cancer [59,60]. In COVID-19, the upregulated activity of ADAM17 increases ACE2 shedding, which in turn leads to higher sACE2 levels [61], and to the increase of TNF-α production. In a kind of positive feedback loop, the ADAM17-mediated shedding activity appears to be enhanced by the binding of the viral spike protein to ACE2 [23,62,63], and to be stimulated by AngII [42] and the pro-inflammatory cytokines interleukin (IL)-1β and TNF-α [64], whose secretion is elevated in COVID-19 patients.

ACE2 shedding can be seen as a sort of defence strategy of the host [48,58]. Indeed, the amount of ACE2 attached to the host cell surface decreases upon shedding and the amount of plasma ACE2 increases accordingly. As both membrane-bound and circulating ACE2 bind to SARS-CoV-2, the net effect of ACE2 shedding is thus the limitation of virus entry into the cells. Moreover, the binding of SARS-CoV-2 spike protein to ACE2 has been suggested to enhance the ACE2 enzymatic activity, and thus to the overactivation of the counter-regulatory ACE2/Ang-(1-7)/MASR axis and to the increase of Ang-(1-7) level in the circulation [65].

Other in vitro and in vivo evidence points to a critical role of circulating sACE2 in SARS-CoV-2 infection. For example, it has been suggested [66] that sACE2 could allow the virus to enter host cells by binding to the SARS-CoV-2 spike protein, alone or in complex with vasopressin, and by performing receptor-mediated endocytosis involving AT1R and the vasopressin receptor AVPR1B, respectively. Note, however, that this mechanism is highly debated in the community and is in contradiction with a number of other evidence [67,68,69]. Indeed, other experiments found that the administration of sACE2 inhibits SARS-CoV-2 infections rather than promoting them [67,68]. Moreover, clinical data show that vasopressin [69] as well as sACE2 [67] infusion in COVID-19 patients does not lead to worsening of the disease, thus suggesting a marginal role of sACE2-mediated viral entry in host cells.

Furthermore, extensive analyses of Prospective Urban Rural Epidemiology (PURE) data, which includes a cohort of about 10,000 patients [70], led to conclude that high levels of plasma ACE2 are strongly associated to an increased risk of major cardiovascular events independently of traditional cardiac risk factors. Other studies also suggest the negative role of high levels of ACE2 and of Ang-(1-7) in cardiovascular diseases [71,72,73,74].

These findings thus point towards an upregulation of the non-classical ACE2/Ang-(1-7)/MASR axis, which is the hallmark of scenario 2. What happens with the classical ACE/AngII/AT1R axis is less clear. It has been argued that it is also upregulated in COVID-19 [58], especially in hypoxic conditions that are common in severe SARS-CoV-2 infection [75]. This suggests a scenario involving the synergistic upregulation of both RAS axes, which would be responsible for the clinical worsening of patient conditions and the severe manifestations of COVID-19.

Finally, other pathways connected to RAS are probably also dysregulated upon SARS-CoV-2 infection. For example, as ACE2 hydrolizes des-Arg9-bradykinin into inactive bradykinin 1–7 peptide, its modulation caused by viral infection could potentially play a role in the dysregulation of the kallikrein-kinin system (KSS) [76]. Given that des-Arg9-bradykinin binds to bradykinin receptor B1, dowregulation of ACE2 is likely to cause an overactivation of this receptor, leading to pulmonary vascular leakage and oedema [77]. The importance of other ACE2-downstream peptides that do not belong to RAS, such as apelins, casomorphins and dynorphins, has also to be investigated, since they could trigger physiological mechanisms worsening COVID-19 patient conditions [78].

## 4. Meta-Analysis on RAS Components in COVID-19

**Figure 3:**
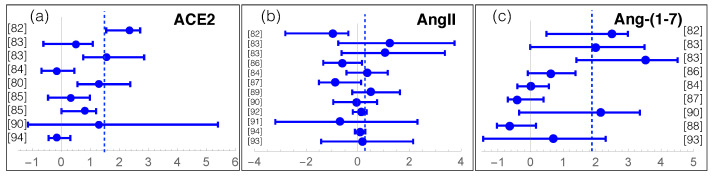
Log-ratios between RAS components (ACE2, AngII and Ang-(1-7)) in COVID-19 patients and controls, with the estimated confidence intervals, for all the collected studies referenced by their reference number (see Table S1). The dashed lines represent the averages of the log-ratios weighted according to the number of patients analyzed in each study. On the y-axis, the reference to the study is mentioned.

We review here recent data about measurements of RAS components in COVID-19. For that purpose, we manually collected and screened a series of clinical and experimental results from [79,80,81,82,83,84,85,86,87,88,89,90,91,92,93,94], listed in Supplementary Table S1. The individuals enrolled in these studies were classified into two or three classes: COVID-19 patients or severe and moderate COVID-19 patients, and controls or asymptomatic patients. We grouped moderate COVID-19 patients and controls into a single class, as their measured values are usually similar. We got thus two classes, which we call (severe) COVID-19 and controls. The average effect of SARS-CoV-2 infection on the levels of RAS components ACE, ACE2, AngI, AngII, Ang-(1-7), Ang-(1-5) is summarized in Table 1 and, for each study, the log-ratio of ACE2, AngII and Ang-(1-7) levels in the COVID-19 and control classes is reported in Figure 3.

The first observation from this meta-analysis is the overactivation of the non-classical RAS axis (Table 1 and Figure 3c). Indeed, the Ang-(1-7) level in COVID-19 patients seems on average more than ten times higher than in controls. Note most studies that analyzed this peptide, involving about 600 patients, show a mild to strong increase in Ang-(1-7). It is instructive to analyze the temporal evolution of Ang-(1-7) level upon infection [83], which seems to remain constant, at a low level, during the whole hospitalization time in non-severe COVID-19 patients. Instead, the Ang-(1-7) level in severe COVID-19 patients is already quite high at hospital entrance, being six times higher than for light COVID-19 patients. Moreover, the level further increases by a factor of five in the first ten days of hospitalization.

Two hypotheses can partially explain the observed Ang-(1-7) upregulation. One is a possible increase of non-ACE2 Ang-(1-7) formation. Indeed, the contribution of a series of peptidases in different tissues may contribute to an increase of the local release of Ang-(1-7) into circulation. For example, Ang-(1-7) can result from the cleavage of AngI via NEP or TO but also from AngII via POP (see Figure 1). This hypothesis is supported by the observation that, in the circulation and in the lungs, the conversion of AngII to Ang-(1-7) is much more POP-dependent than ACE2-dependent [95]. Note, however, that in a recent investigation [93] no difference was observed between POP activity in COVID-19 patients and controls.

A second hypothesis relates Ang-(1-7) upregulation to an increase of ACE2 shedding from the cell membrane. Indeed, as shown in Table 1 and Figure 3a, the levels of soluble, circulating, sACE2, averaged over the set of more than thousand collected samples, are 1.5 higher in COVID-19 patients than in controls. Some studies, which found that elevated sACE2 plasma levels is associated with a worse clinical outcome, suggested its use as a clinical marker of the infection [85].

The increase of sACE2 is, moreover, associated with an equally massive increase of its activity. Indeed, a 20-fold increase of ACE2 activity from the time of admission in the hospital, and a 40-fold increase compared to the values of controls, was observed in a case report [96]. In a more systematic analysis of more than 130 patients (66 COVID-19 and 70 controls) [81], a factor of almost 100 between controls and COVID-19 patients is observed, with ACE2 activity values of 0.06 pmol/min/mL and 5.8 pmol/min/mL, respectively. Note that ACE2 activity remains quite elevated up to a median of 114 days post infection [81].

More debated is the role of AngII. The data collected shows a slightly increased average level of AngII in COVID-19 patients with respect to controls (Table 1). More precisely, roughly half of the studies observe a decrease in AngII levels [82,86,87] and the others, an increase [83,84,89] (Figure 3b). Instead, one would naively expect a general decrease of AngII level, given that ACE2 shows upregulated activity and cleaves AngII. The AngII increase observed in some studies is even more surprising considering that the average ACE and AngI levels are decreased (see Table 1), which is expected to further contribute to a decrease in AngII level, as it is the catalytic product of ACE acting on AngI. Note that the AngII levels have been suggested to change substantially according to the stage of the viral infection [58]. This could partially explain the AngII variability reported in Table 1, even though the full understanding of the AngII levels and their evolution in COVID-19 remains elusive without additional data.

The observed overactivation of the non-classical RAS axis is a priori expected to yield an increase of circulating Ang-(1-5), obtained by cleavage of Ang-(1-7) by ACE (Figure 1). However, this is only the case if we assume that ACE activity remains constant during viral infection. Indeed, a strong decrease in ACE activity could balance the increase in Ang-(1-7) level and lead to a constant or even a decrease of the circulating Ang-(1-5). As a matter of fact, contrasting results were found for ACE activity [79,90,93]. An investigation found significantly lower activity in COVID-19 patients with respect to controls [79], while other studies found opposite trends [90] or no differences [93]. Opposite results were also observed for the levels of circulating Ang-(1-5) in two studies: a reduction of about 80% in the COVID-19 class with respect to controls [86], or a 100% increase [82] (see Supplementary Table S1).

A possible explanation of these contradictory observations is that some of the endogenous levels of RAS components analyzed here were obtained using commercial immunoessay (ELISA) kits, as specified in Supplementary Table S1. However, these kits do not have sufficient sensitivity to accurately determine the low peptide concentrations of RAS components, which are in the range of pg/mL [97]. Valid alternatives for peptide quantification, such as mass spectroscopy combined with ultrahigh-pressure liquid chromatography (LC-MS), do exist but are more complex, lab-time consuming and expensive. It should be noted, however, that the general trends remain the same if we exclude the measurements carried out with ELISA kits, and thus that these do not explain all contradictory and unexpected observations.

## 5. RAS-Targeting Drugs in COVID-19

Due to the role that RAS has in SARS-COV-2 infection, multiple RAS-targeting drugs have been proposed to alleviate the severity of COVID-19 clinical manifestations. Here we briefly review some of them, focusing in particular on human recombinant ACE2 (rhACE2) and the exogenous administration of Ang-(1-7). Extensive meta-data analyses and reviews of the role of common RAS-blockers in COVID-19, such as ACE inhibitors or angiotensin receptor blockers, have recently been published and we thus refer the reader to these works [98,99,100].

In light of the first scenario in which the RAS imbalance shown in Figure 2b is responsible of disease exacerbation, an appealing way to restore RAS functionality would be to deliver rhACE2 that could act with a double role of binding to the spike protein of the viral particle, thus slowing down the viral infection, but also downregulating AngII and boosting the non-classical RAS axis, thereby protecting the lungs from injury.

It seems established through a series of studies, which even pre-date the SARS-CoV-2 pandemic, that administration of rhACE2 to animal models affected by ARDS appears to reverse the lung-injury process. Examples of improvement have been observed in acute lung injury induced by sepsis [34], SARS-CoV spike protein [34], H5N1 [101] and syncytial virus infection [102]. More recently, rhACE2 has been shown to bind with high affinity also to the SARS-CoV-2 spike protein, to be capable of inhibiting the attachment of this virus to the host cells and thus to neutralize it at least in vitro [68,103]. In another in vitro study [104], rhACE2 in combination with remdesivir, an antiviral medication, has proven to boost their antiviral efficacies.

Phase I and II clinical trials of administration of rhACE2 have been performed to exploit and test its ability to ameliorate ARDS in humans [105,106]. rhACE2 treatment has been proven to be well tolerated without major adverse effects in these pilot studies. However, despite the significant AngII level reduction accompanied by the rapid upregulation of RAS peptides Ang-(1-7) and Ang-(1-5), no significant impact in the alleviation of the clinical severity of ARDS has been observed, with no difference in PaO${}_{2}$/FiO${}_{2}$ (partial pressure of arterial oxygen to fraction of inspired oxygen) ratio between treatment groups and placebo [106].

In a recent case study, a SARS-CoV-2 patient was administered twice daily with rhACE2 intravenous infusion (0.4 mg/kg) [67]. The trends were similar to those observed in [106], namely a reduction of AngII and an increase of Ang-(1-7) and Ang-(1-5) levels. A large phase II clinical trial (NCT04335136) has been initiated in April 2020 but no public results are available at the moment. Further analyses and data are needed to better understand the biological differences between the effect of rhACE2 responses in animal models, where an improvement of the lung-injury is usually observed, and in humans, where no substantial improvement of the clinical condition is visible in the current studies.

There are some drawbacks to using rhACE2, such as its short half-life in plasma [107]. It has for example be claimed [106] that a continuous infusion of rhACE2 should be used to reach a higher efficiency, and that no effect has been seen in humans for this reason. Protein stabilization methods such as chimeric fusion of rhACE2 with IgG2 Fc fragments have been shown to improve rhACE2 stability in plasma and could lead to an improved treatment effectiveness not only in SARS-CoV-2 but also in a series of RAS-related disorders [108,109].

Another therapeutic approach consists in using ACE2-derived peptides that are expected to have improved pharmacokinetic and pharmacodynamic profiles with respect to rhACE2. These peptides could be administered via inhalation and, similarly to rhACE2, they could contribute to slow down the viral infection and inhibit the local secretion of pro-inflammatory agents [110]. Despite the efforts towards the computational design of such peptides [111,112], their development is still in an initial phase and data about their efficacy, from animal models or clinical trials, are lacking.

Preclinical evidence suggests that not only rhACE2 but also the exogenous administration of Ang-(1-7) peptide can improve severe clinical ARDS conditions such as low oxygenation and high inflammation status [113,114]. Thus, administration of Ang-(1-7) or Ang-(1-7) agonists has been suggested to mitigate SARS-CoV-2 infections by boosting the anti-inflammatory response via MASR activation [10,115].

Several clinical trials of phase I and II are currently ongoing to assess the safety of the administration of Ang-(1-7) and its possible effects on the clinical manifestations of COVID patients (NCT04375124, NCT04633772, NCT04605887, NCT04570501, NCT04401423). However, the trials are still in an early stage at the moment and there are no indications about the effectiveness of Ang-(1-7) in COVID-19. Note that not only Ang-(1-7) but also some of its agonists or stabilized versions, such as the protease-resistant cyclic form of Ang1–7 [cAng1–7], which binds to MASR, could equally stimulate anti-inflammatory downstream signaling [114,116,117]. They could represent a valid alternative to be administrated in severe COVID-19 patients since they are characterized by an increased stability with respect to linear Ang-(1-7) peptides that are known to have a very short half-life [118,119].

Consider now scenario 2 of dysregulated RAS represented in Figure 2c, in which the two RAS axes are overactivated. At first sight, normal RAS functioning could be restored in this scenario by acting against the peak of ACE2 activity. Indeed, inhibiting ACE2 or the metalloprotease ADAM17 that is responsible for ACE2 shedding could be an effective way to block the overactivation of the RAS axes, which in turn would lead to the suppression of the inflammatory responses in COVID-19 and attenuate severe lung injury [61,120]. Some studies propose the introduction of ACE2 inhibitor drugs [57]. Since ACE2 is a zinc-metalloprotease, the use of zinc-chelating agents is proposed, such as citrate and ethylenediaminetetraacetic acid (EDTA), alone or in combination, to reduce the protein’s activity and decrease the hypothesized positive feedback loop of the non-classical RAS axis.

One chelating agent tested in SARS-CoV-2 infection is ranitidine bismuth citrate, which is usually used in the treatment of *helicobacter pylori* infection [121]. It is effective in rescuing animal models from SARS-CoV-2-induced pneumonia by suppressing viral load. Even though ranitidine bismuth citrate can target zinc-containing proteins and thus ACE2 and ADAM17, the authors of this study suggest that the observed mitigation of SARS-CoV-2 disease upon its administration is related to its action on the viral helicase, thus impeding SARS-CoV-2 efficient replication [121]. There is thus no connection between this drug and RAS dysregulation at the moment.

A last natural strategy in the context of scenario 2 to reduce ACE2 overactivity, responsible for the exacerbated inflammatory responses in COVID-19, is to target ACE2 shedding via the inhibition of ADAM17 [120]. Two ADAM17 inhibitors have been tested in animal models of COVID-19, apratastat (TMI-005) and TMI-1, resulting in a significant reduction in the production of pro-inflammatory cytokines as well as in protective effects against lung injury associated with COVID-19 [122].

## 6. Discussion

RAS undoubtedly plays important roles in the regulation of many physiological and pathophysiological processes in humans and gets dysregulated upon SARS-CoV-2 infection. However, the reason why and the way in which RAS is dysregulated in COVID-19 is currently the subject of intense debate. The meta-analysis that we carried out in the previous section has shed some light on these questions to a certain extent, but many points remain obscure. Indeed, the experimental and clinical data that we collected from the literature about e.g., the levels of proteins and peptides involved in RAS and the activity of the enzymes, partially disagree [79,80,81,82,83,84,85,86,87,88,89,90,91,92,93,94].

Recent experimental data point to an overactivation of the non-classical RAS axis ACE2/Ang-(1-7)/MASR, with an increase of the level of circulating Ang-(1-7) and a concomitant increase of serum ACE2 activity, in contrast with earlier scenarios [24,45,46] which rather suggested a downregulation of this axis (see Figure 2). These observations possibly result from increased shedding of ACE2 from the membrane and thus to the release in the serum of the catalytically active ACE2 ectodomain. Experimental evidence suggests that ACE2 activity is moreover increased due to the conformational modifications that occur upon binding to the receptor binding subunit S1 of the viral spike protein [65]. Another possible reason of the increase in Ang-(1-7) level in COVID-19 is its formation via non-ACE2 pathways, i.e., through the cleavage of AngI via NEP or TO and of AngII via POP, especially since the latter pathway has been suggested as dominant for AngII to Ang-(1-7) conversion in lungs and the circulating system [95].

There is an apparent discrepancy between the known protective role of cell-bound ACE2 and the negative COVID-19 outcome upon upregulation of the ACE2/Ang-(1-7)/MASR axis. The increase of circulating Ang-(1-7) and ACE2 levels and of ACE2 activity could reflect a reduced local, pulmonary, ACE2 enzymatic activity associated to a local increase of AngII levels upon SARS-CoV-2 infection. Note that the reduction of local ACE2 could be further worsened by AngII-mediated ACE2 internalization [123], thus acting as a sort of feedforward loop in which reducing ACE2 increases the level of AngII which in turn decreases ACE2.

The biological interpretation of RAS dysregulation in COVID-19 is thus highly complex. It requires a clear distinction between tissue and plasma ACE2, which is not obvious to measure [70,124]. While it is quite clear that cell-bound ACE2 and the counter-regulatory ACE2/Ang-(1-7)/MASR axis plays a protective role in inflammation and cardiovascular diseases [125], elevated ACE2 levels in plasma rather seem to a have a negative impact and could be indirectly related to an overactivation of the classical ACE/AngII/AT1R axis in the local RAS.

Moreover, the complex interactions between ACE2 cellular expression, internalization, enzymatic cleavage and plasma clearance are not yet understood [70] and certainly deserve further analyses to better understand how RAS alterations affect COVID-19 severity. For example, ACE2 in its membrane-bound form represent about 97–98% of the total amount of ACE2 in healthy individuals, while ACE2 in its circulating, soluble, form represents only 2–3% [63,90]. How this disproportion gets modified in COVID-19 is not known, but an increase in circulating ACE2 (see Table 1) could be insufficient to counterbalance the downregulation of the local ACE2 in the lungs or the heart. The picture is further complicated by the different roles and expression levels that ACE2 has in different tissues.

It is also interesting to understand why the increase in Ang-(1-7) (Table 1) is not sufficient to trigger the anti-inflammatory and anti-fibrotic response, although it is sufficient in animal models and in other pathological conditions [9,10]. This could be due to a variety of factors such as the insufficient levels of global Ang-(1-7), but also to its plasma clearance or very short half-life. The role of MASR and its expression should also be investigated to better understand this point, since MASR downregulation could lead to the downregulation of the downstream signaling pathway even in the presence of an increased Ang-(1-7) level.

Finally, another hypothesis that cannot be totally ruled out is that RAS dysregulation in COVID-19 is not caused directly by the viral infection but rather is associated with severe events that occur in acute forms of COVID-19. To explore this hypothesis in more detail, it is crucial to define the matched COVID-19 negative control group in the analysis of RAS peptide levels. For example, severe COVID-19 patients were compared with influenza patients presenting similar disease severity, and with ARDS patients without COVID-19, respectively [83,93]. Unfortunately, the results from these two studies disagree: the former study observed significant differences and the latter not.

In summary, despite the numerous studies that hypothesize a relation between RAS dysregulation and SARS-CoV-2 infection, this relation is not yet clearly understood and remains to be deciphered. More clinical, experimental and computational data are needed to further shed light on this issue and to identify RAS-targeted therapeutic strategies that are able to mitigate severe COVID-19 clinical manifestations.

## Figures and Tables

**Table 1:** Average change (in %) of the serum concentration of RAS components between COVID-19 patients and controls. The averages were performed over all the studies collected in this paper and weighted according to the number of patients/controls in each study. *p*-Values were computed using the Stouffer method and are reported only when statistically significant (p≤ 0.05). *N* is the total number of patients and controls. Increased levels are indicated on a pink background. See Supplementary Table S1 for the exact values in each cohort.

RAS Component	COVID-19 Effect	*p*-Value	*N*
ACE	−10%	-	124
ACE2	+158%	<$10^{-8}$	1024
AngI	−22%	-	263
AngII	+74%	∼0.05	840
Ang-(1-7)	+1100%	<$10^{-6}$	602
Ang-(1-5)	−28%	-	53

## Supplementary Materials

The following are available online at https://www.mdpi.com/article/10.3390/cells10102755/s1, Table S1: Mean RAS peptide levels and enzymatic activities collected from dierent studies.
