# Supplementary material for: Quantifying Renin-Angiotensin-System Alterations in COVID-19
Source: Cells. 2021 Oct 14;10(10):2755. doi: 10.3390/cells10102755 (PMC8535134; doi:10.3390/cells10102755)
Supplement: Supplementary file 1 [file cells-10-02755-s001.zip › cells-1395009-supplementary.pdf]

Supplementary Material

Quantifying Renin-Angiotensin-System Alterations in COVID-19

F. Pucci, F. Annoni, R.A. Santos, F.S. Taccone, M. Rooman

Table 1. Mean RAS peptide levels and enzymatic activities collected from different studies.

	Unit	Severe	Non-Severe	Controls	*p*-value	*N*	Method	Ref
ACE activity	U/mL	49 [37–68]	64 [52–78]	75 [58–99]	<0.001	196	KPSM	[1]
	U/mL	44 [32–53]	48 [36–55]	35 [25–51]	0.01	96	ELISA	[2]
	RFU/nL	0.22 [0.27–0.18]	0.2 [0.3–0.16]	0.3 [0.35–0.24]	-	33	FA	[3]
ACE level	ng/mL	57 [45–70]		64 [48–265]	-	124	ELISA	[4]
ACE2 activity	RFU/nL	0.15 [0.2–0.09]	0.16 [0.2–0.11]	0.13 [0.16–0.1]	-	33	FA	[3]
	pmol/min/mL	11.2 [8.3–22]	5.4 [1.8–9.0]	0.06 [0.02–2.2]	<0.0001	136	FA	[5]
POP activity	RFU/nL	0.09 [0.13–0.06]	0.12 [0.07–0.17]	0.08 [0.12–0.05]	-	33	FA	[3]
ACE2 level	ng/mL	8.5 [4.7–10.6]		0.8 [0.7–1.0]	<0.001	15	LC-MS	[6]
	ng/mL	2.1 [1.3–3.0]	1.3 [1.0–2.4]	-	-	153	LC-MS	[7]
	ng/mL	15.1 [9.8–32]	3.2 [1.8–4.7]	-	<0.001	153	LC-MS	[7]
	ng/mL	19.4 [±2]		22.6 [±4]	<0.05	45	ELISA	[8]
	ng/mL	5.0 [2.8–11.8]		1.4 [1.1–1.6]	<0.0001	124	ELISA	[4]
	NPX	1.5 [1.0–2.4]	1.1 [0.9–1.6]		<0.001	205	PEA	[9]
	NPX	4 [2.4–5.0]	1.8 [1.5–2.4]		<0.0001	205	PEA	[9]
	ng/mL	1.4 [0.7–3.5]	2.1 [0.6–22.4]	0.5 [0.1–1.9]	0.001	96	ELISA	[2]
	ng/mL	1.3 [±0.1]	1.5 [±0.1]	-	<0.01	85	ELISA	[10]
AngI level	ng/mL	94 [46–300]		43 [37–95]	-	15	LC-MS	[6]
	ng/mL	31 [±6]		41 [±] 10	-	38	LC-MS	[11]
	ng/mL	1.5 [1.3–2.0]		3.3 [2.0–4.0]	<0.005	45	ELISA	[8]
	ng/mL	0.4 [0.4–0.5]	1.0 [0.6–1.5]	-	0.03	28	ELISA	[12]
	pg/mL	42 [32–45]	39 [26–47]	37 [24–47]	-	96	ELISA	[2]
	ng/mL	0.15 [0.1–0.22]		0.7 [0.5–1.0]	<0.001	41	ELISA	[13]
AngII level	ng/mL	52 [30–78]		137 [105–495]	0.008	15	LC-MS	[6]
	pmol/L	166 [61–680]	48 [16–131]	-	<0.01	153	LC-MS	[7]
	pmol/L	96 [38–250]	33 [9–78]	-	<0.01	153	LC-MS	[7]
	ng/mL	6.0 [±1.1]		10.7 [±1.9]	<0.05	38	LC-MS	[11]
	ng/mL	1.0 [0.7–1.3]		0.7 [0.4–1.1]	<0.05	45	ELISA	[8]
	ng/mL	0.25 [0.2–0.3]	0.6 [0.25–0.9]	-	0.04	28	ELISA	[12]
	ng/mL	0.21 [0.13–0.42]		0.13 [0.08–0.17]	<0.01	27	ELISA	[14]
	pg/mL	56 [23–131]	99 [65–153]	103 [71–140]	-	94	ELISA	[2]
	ng/mL	0.15 [0.13–0.17]	0.13 [0.12–0.16]	-	<0.01	55	ELISA	[15]
	ng/mL	0.4 [0.2–3.0]		0.8 [0.3–4.8]	<0.001	112	ELISA	[16]
	ng/mL	4.8 [4.4–5.2]		4.4 [4.2–4.6]	<0.01	85	ELISA	[10]
	pmol/L	25 [14–35]	18 [11–25]	17 [8–25]	-	33	RIA	[3]
Ang1–7 level	ng/mL	24 [5.4–40]		2.0 [2.0–3.3]	0.004	15	LC-MS	[6]
	pmol/L	11 [5–51]	1.5[1.5–5.0]	-	<0.001	153	LC-MS	[7]
	pmol/L	50 [15–132]	1.5 [1.5–3.7]	-	<0.001	153	LC-MS	[7]
	ng/mL	14 [±2.3]		7.5 [±1.4]	<0.05	38	LC-MS	[11]
	ng/mL	0.5 [0.4–0.7]		0.5 [0.4–0.6]	-	45	ELISA	[8]
	ng/mL	0.2 [0.2–0.3]	0.3 [0.2–0.4]	-	0.04	28	ELISA	[12]
	pg/mL	14.3 [1.6–33]	8.4 [2.8–28]	0.98 [0.98–3.7]	<0.001	96	ELISA	[2]
	ng/mL	0.17 [0.13–0.27]		0.32 [0.22–0.37]	0.003	41	ELISA	[13]
	pmol/L	15 [0–60]	17 [0–70]	10 [5–15]	-	33	RIA	[3]
Ang1–5 level	ng/mL	9.0 [5.7–14]		4.3 [2.6–14]	-	15	LC-MS	[6]
	ng/mL	3.4 [±0.8]		19.3 [±6.3]	<0.01	38	LC-MS	[11]

The values for three classes of individuals (severe COVID-19, non-severe COVID-19 and controls) or two classes (COVID-19 and controls) are reported. *N* is the number of samples on which the experimental measures have been done. The different experimental methods used in the measurements of the peptide levels are: liquid chromatography-mass spectrometry (LC-MS), enzyme–linked immunosorbent assay (ELISA), kinetic spectrophotometry (KPSM), fluorescence assay

(FA), radioimmunoassay (RIA) and proximity extension assay (PEA). Only the *p*-values reported by the authors to be statistically significant (<0.05) are shown.

References

1. Zhu, Z.; Cai, T.; Fan, L.; Lou, K.; Hua, X.; Huang, Z; Gao, G. The potential role of serumangiotensin-converting enzyme in coronavirus disease 2019. *BMC Infect. Dis.* **2020**, *20*, 883.
2. Burns, K.; Cheng, M.; Lee, T.; McGeer, A.; Sweet, D.; Tran, K.; Lee, T.; Murthy, S.; Boyd, J.; Singer, J.; et al. Sustained dysregulation of the plasma renin–angiotensin system in acute COVID-19. *Research Square* **2021**, in preprint.
3. Files, D.C.; Gibbs, K.W.; Schaich, C.L.; Collins, S.P.; Gwathmey, T.M.; Casey, J.D.; Self, W.H.; Chappell, M.C. A Pilot Study to Assess the Circulating Renin-Angiotensin-System in COVID-19 Acute Respiratory Failure. *Am. J. Physiol. Cell. Mol. Physiol.* **2021**, *321*, L213–L218, doi:10.1152/ajplung.00129.2021.
4. Lundström, A.; Ziegler, L.; Havervall, S.; Rudberg, A.S.; von Meijenfeldt, F.; Lisman, T.; Mackman, N.; Sandén, P.; Thålin, C. Soluble angiotensin–converting enzyme 2 is transiently elevated in COVID–19 and correlates with specific inflammatory and endothelial markers. *J.Med. Virol* **2021**,*93*, 5908–5916.
5. Patel, S. K.; Juno, J. A.; Lee, W. S.; Wragg, K. M.; Hogarth, P. M.; Kent, S. J.; Burrell, L. M. Plasma ACE2 activity is persistently elevated following SARS-CoV-2 infection: implications for COVID-19 pathogenesis and consequences. *Eur. Respir. J.* **2021**, *57*, 2003730.
6. van Lier, D.; Kox, M.; Santos, K.,; van der Hoeven, H.; Pillay, J.; Pickkers, P. Increased blood angiotensin converting enzyme 2 activity in critically ill COVID-19 patients. *ERJ. Open Res.* **2021**, *7*, 00848–02020.
7. Reindl-Schwaighofer, R.; Ho¨dlmoser, S.; Eskandary, F.; Poglitsch, M.; Bonderman, D.; Strassl, R.; Aberle, J. H.; Oberbauer, R.; Zoufaly, A.; Hecking, M. ACE2 elevation in severe COVID-19. *Am J Respi.r Crit. Care Med.* **2021**, *203*, 1191–1196.
8. Osman, I.O.; Melenotte, C.; Brouqui, P.; Million, M.; Lagier, J.-C.; Parola, P.; Stein, A.; La Scola, B.; Meddeb, L.; Mege, J.-L.; et al. Expression of ACE2, Soluble ACE2, Angiotensin I, Angiotensin II and Angiotensin-(1-7) Is Modulated in COVID-19 Patients. *Front. Immunol.* **2021**, *12*, 625732, doi:10.3389/fimmu.2021.625732.
9. Kragstrup, T.W.; Singh, H.S.; Grundberg, I.; Nielsen, A.L.-L.; Rivellese, F.; Mehta, A.; Goldberg, M.B.; Filbin, M.R.; Qvist, P.; Bibby, B.M. Plasma ACE2 predicts outcome of COVID-19 in hospitalized patients. *PLoS. ONE.* **2021**, *16*, e0252799, doi:10.1371/journal.pone.0252799.
10. Rieder, M.; Wirth, L.; Pollmeier, L.; Jeserich, M.; Goller, I.; Baldus, N.; Schmid, B.; Busch, H.-J.; Hofmann, M.; Kern, W.; et al. Serum ACE2, Angiotensin II, and Aldosterone Levels Are Unchanged in Patients With COVID-19. *Am. J. Hypertens.* **2021**, *34*, 278–281, doi:10.1093/ajh/hpaa169.
11. Martins, A. L. V.; da Silva, F. A.; Bolais-Ramos, L.; de Oliveira, G. C.; Ribeiro, R. C.; Pereira, D. A. A.; D., Annoni, F.; Diniz, M. M. L.; Silva, T. G. F.; Increased circulating levels of angiotensin-(1–7) in severely ill COVID-19 patients. *ERJ. Open. Res.* **2021**, *7*, 00114–02021.
12. Eleuteri, D.; Montini, L.; Cutuli, S.L.; Rossi, C.; Alcaro, F.; Antonelli, M. Renin–angiotensin system dysregulation in critically ill patients with acute respiratory distress syndrome due to COVID-19: a preliminary report. *Critical Care* **2021**, *25*, 91.
13. Henry, B.M.; Benoit, J.L.; Berger, B.A.; Pulvino, C.; Lavie, C.J.; Lippi, G.; Benoit, S.W. Coronavirus disease 2019 is associated with low circulating plasma levels of angiotensin 1 and angiotensin 1,7. *J. Med Virol.* **2021**, *93*, 678–680, doi:10.1002/jmv.26479.
14. Wu, Z.; Hu, R.; Zhang, C.; Ren, W.; Yu, A.; Zhou, X. Elevation of plasma angiotensin II level is a potential pathogenesis for the critically ill COVID-19 patients. *Crit. Care* **2020**, *24*, 1–3, doi:10.1186/s13054-020-03015-0.
15. Liu, N.; Hong, Y.; Chen, R.-G.; Zhu, H.-M. High rate of increased level of plasma angiotensin ii and its gender difference in covid–19: an analysis of 55 hospitalized patients with COVID-19 in a single hospital, wuhan, china. *medRxiv* **2020**, in preprint..
16. Ozkan, S.; Cakmak, F.; Konukoglu, D.; Biberoglu, S.; Ipekci, A.; Akdeniz, Y.S.; Bolayirli, I.M.; Balkan, I.I.; Dumanli, G.Y.; Ikizceli, I. Efficacy of Serum Angiotensin II Levels in Prognosis of Patients With Coronavirus Disease 2019. *Crit. Care Med.* **2021**, *49*, e613–e623, doi:10.1097/ccm.0000000000004967.
